# New insights into the development of the human cerebral cortex

**DOI:** 10.1111/joa.13055

**Published:** 2019-08-02

**Authors:** Zoltán Molnár, Gavin J. Clowry, Nenad Šestan, Ayman Alzu'bi, Trygve Bakken, Robert F. Hevner, Petra S. Hüppi, Ivica Kostović, Pasko Rakic, E. S. Anton, David Edwards, Patricia Garcez, Anna Hoerder‐Suabedissen, Arnold Kriegstein

**Affiliations:** ^1^ Department of Physiology, Anatomy and Genetics University of Oxford Oxford UK; ^2^ Institute of Neuroscience Newcastle University Newcastle upon Tyne UK; ^3^ Department of Neuroscience, Yale University School of Medicine New Haven CT USA; ^4^ Department of Basic Medical Sciences Faculty of Medicine Yarmouk University Irbid Jordan; ^5^ Allen Institute for Brain Science Seattle WA USA; ^6^ Department of Pathology UC San Diego La Jolla CA USA; ^7^ Dept. de l'enfant et de l'adolescent Hôpitaux Universitaires de Genève Genève Switzerland; ^8^ Croatian Institute for Brain Research School of Medicine University of Zagreb Zagreb Croatia; ^9^ UNC Neuroscience Center Department of Cell and Molecular Physiology The University of North Carolina School of Medicine Chapel Hill NC USA; ^10^ Centre for the Developing Brain Biomedical Engineering and Imaging Sciences, King's College London London UK; ^11^ Federal University of Rio de Janeiro, UFRJ Institute of Biomedical Sciences Rio de Janeiro Brazil; ^12^ Department of Neurology University of California, San Francisco (UCSF) San Francisco CA USA; ^13^ The Eli and Edythe Broad Center of Regeneration Medicine and Stem Cell Research UCSF San Francisco CA USA

**Keywords:** associative areas, calretinin, GABA, inhibitory interneurons, neurogenesis, neuroimaging, neuronal progenitors, prefrontal cortex, subplate neurons

## Abstract

The cerebral cortex constitutes more than half the volume of the human brain and is presumed to be responsible for the neuronal computations underlying complex phenomena, such as perception, thought, language, attention, episodic memory and voluntary movement. Rodent models are extremely valuable for the investigation of brain development, but cannot provide insight into aspects that are unique or highly derived in humans. Many human psychiatric and neurological conditions have developmental origins but cannot be studied adequately in animal models. The human cerebral cortex has some unique genetic, molecular, cellular and anatomical features, which need to be further explored. The Anatomical Society devoted its summer meeting to the topic of Human Brain Development in June 2018 to tackle these important issues. The meeting was organized by Gavin Clowry (Newcastle University) and Zoltán Molnár (University of Oxford), and held at St John's College, Oxford. The participants provided a broad overview of the structure of the human brain in the context of scaling relationships across the brains of mammals, conserved principles and recent changes in the human lineage. Speakers considered how neuronal progenitors diversified in human to generate an increasing variety of cortical neurons. The formation of the earliest cortical circuits of the earliest generated neurons in the subplate was discussed together with their involvement in neurodevelopmental pathologies. Gene expression networks and susceptibility genes associated to neurodevelopmental diseases were discussed and compared with the networks that can be identified in organoids developed from induced pluripotent stem cells that recapitulate some aspects of *in vivo* development. New views were discussed on the specification of glutamatergic pyramidal and γ‐aminobutyric acid (GABA)ergic interneurons. With the advancement of various *in vivo* imaging methods, the histopathological observations can be now linked to *in vivo* normal conditions and to various diseases. Our review gives a general evaluation of the exciting new developments in these areas. The human cortex has a much enlarged association cortex with greater interconnectivity of cortical areas with each other and with an expanded thalamus. The human cortex has relative enlargement of the upper layers, enhanced diversity and function of inhibitory interneurons and a highly expanded transient subplate layer during development. Here we highlight recent studies that address how these differences emerge during development focusing on diverse facets of our evolution.

## Introduction

There has undoubtedly been a great leap forward in the evolution of cognitive processing in the human brain as compared with the brains of other mammalian species, but where does this evolutionary boost come from? What, if any, are the fundamental differences in the neural circuitry of the human cerebral cortex and associated structures that make us different from the mouse, our currently favored model organism, or even other primates? Are we merely blessed with a relatively larger cortex giving us greater processing power, or are there specific enhancements in the way our neural circuits are organized? As gene expression changes during development have the greatest capacity to drive macro‐evolutionary changes (Carroll, 2008), it is productive to search for specific differences in developmental processes in the cerebral cortex, compared with other species, that might give rise to our enhanced cognitive powers (Rakic, 2009; Kaas, 2013).

In primates, higher order associative areas have greatly increased in size and complexity (Uylings & van Eden, 1991; Molnár & Pollen, 2014). Two associative areas, frontal and parietal, were identified as unique to, or at least more highly developed in, the primate cortex; the frontal associative (prefrontal) area is the largest and covers the anterior part of the frontal lobe (almost covering 80% of the entire frontal lobe) and one‐third of the total cortical surface (Teffer & Semendeferi, 2012; Hladnik et al. 2014). It is considered the key element for highest‐order cognitive functions in humans, such as working memory, language, decision‐making and social behavior (O'Rahilly & Müller, 2008; Friederici, 2011; Roth & Dicke, 2012; Teffer & Semendeferi, 2012). The deep layer III pyramidal neurons in these associative areas form crucial elements for substantial numbers of connections with other cortical areas (Barbas et al. 2005; Yeterian et al. 2012), which are essential substrates involved in higher cognitive abilities (Selemon et al. 2003; Wang et al. 2006; Verduzco‐Flores et al. 2009). In addition to cortical expansion and vast cortical‐cortical connectivity, the intrinsic organization of cortical circuitry has also been a target for evolutionary adaptation related to higher cortical function in primates (Burkhalter, 2008; Forbes & Grafman, 2010). The efficiency of cortical circuitry is highly dependent on the function of inhibitory γ‐aminobutyric acid (GABA)ergic interneurons, which act as intrinsic modulators essential to higher order processing (Whittington et al. 2011; Buzsáki & Wang, 2012), and may themselves be subject to evolutionary processes or events unique to certain mammalian families (Sousa et al. 2017).

Mice have been the model of choice because of historic reasons, in large part due to their genetic tractability (Goffinet & Rakic, 2000; Molnár & Price, 2016). There are shared and distinct features of mouse and human brain development (Clowry et al. 2010; Molnár & Clowry, 2012). Over the course of development, both species undergo similar cellular processes, including cell proliferation and migration, and a comparable temporal order of events. Mouse and human have homologous cell types and, in many cases, utilize identical molecular programs. Further, mice and humans are posited to have similar plasticity mechanisms and elucidating basic principles of development and circuitry, the mouse remains extremely valuable. While studies in rodents have been incredibly useful in teasing apart aspects of the cerebral cortical development, it is important to appreciate that these observations cannot simply be transferred to humans. Mouse cortex is the product of its own unique evolutionary forces that resulted in a small body and lissencephalic brain, a non‐laminated lateral geniculate nucleus, and lateral‐set eyes with minimal binocular vision. There are clear gene expression and cellular differences even between mouse and rat, but the genetic underpinnings of their divergent evolution are still not understood (Silver et al. 2019). We are only at the very beginning of defining aspects of cortical development that are mouse specific and those that are human specific and those that are shared across the mammals. Our ultimate goal is to reveal cortical differences that distinguish humans from other species and understand how these features parlay into unique human behavior (Geschwind & Rakic, 2013; Molnár & Pollen, 2014). Relative to non‐human primates, human possess a higher brain to body ratio, more neurons, and a greater degree of brain lateralization (Silver et al. 2019). The human brain has more neurons, neuronal diversity and morphological differences are larger in human, enlarged supragranular layers, non‐cortical structures also evolved, such as cerebellum, higher order nuclei within the thalamus are massively enlarged in primates and mediate transthalamic cortico‐cortical interactions. The more complex human brain develops during a longer gestational period and has a more extended adolescence. Study of human and various animal models using identical techniques such as comparative single‐cell transcriptomics, magnetic resonance imaging (MRI) or electrophysiology could be valuable to compare cortical areas between humans and animal models even if such attempts come with challenges (Silver et al. 2019).

Understanding human cortical development should also help us to understand, and ultimately treat, a number of neurodevelopmental conditions that arise from genetic or environmental insults to the developing brain. Thus, this review will consider advances made in combining genome‐wide association with gene expression studies to try and elucidate the role of autism and schizophrenia susceptibility genes in normal development in order to model the role of mutations in causing disability. Pre‐ and perinatal imaging provides more detailed information on the development of the subplate, and the effects of prematurity and hypoxia. Finally, the emergence of human cerebral organoids as a developmental model has shown great utility in mimicking human neurodevelopmental disease, leading to an understanding of why Zika virus (ZIKV) infection *in utero* may cause microcephaly, and how it might be treated, following the recent outbreak around the world and especially in South America (Gilbert‐Jaramillo et al. 2019).

## Neurogenesis, diversification of radial glia subtypes

**Figure 1:**
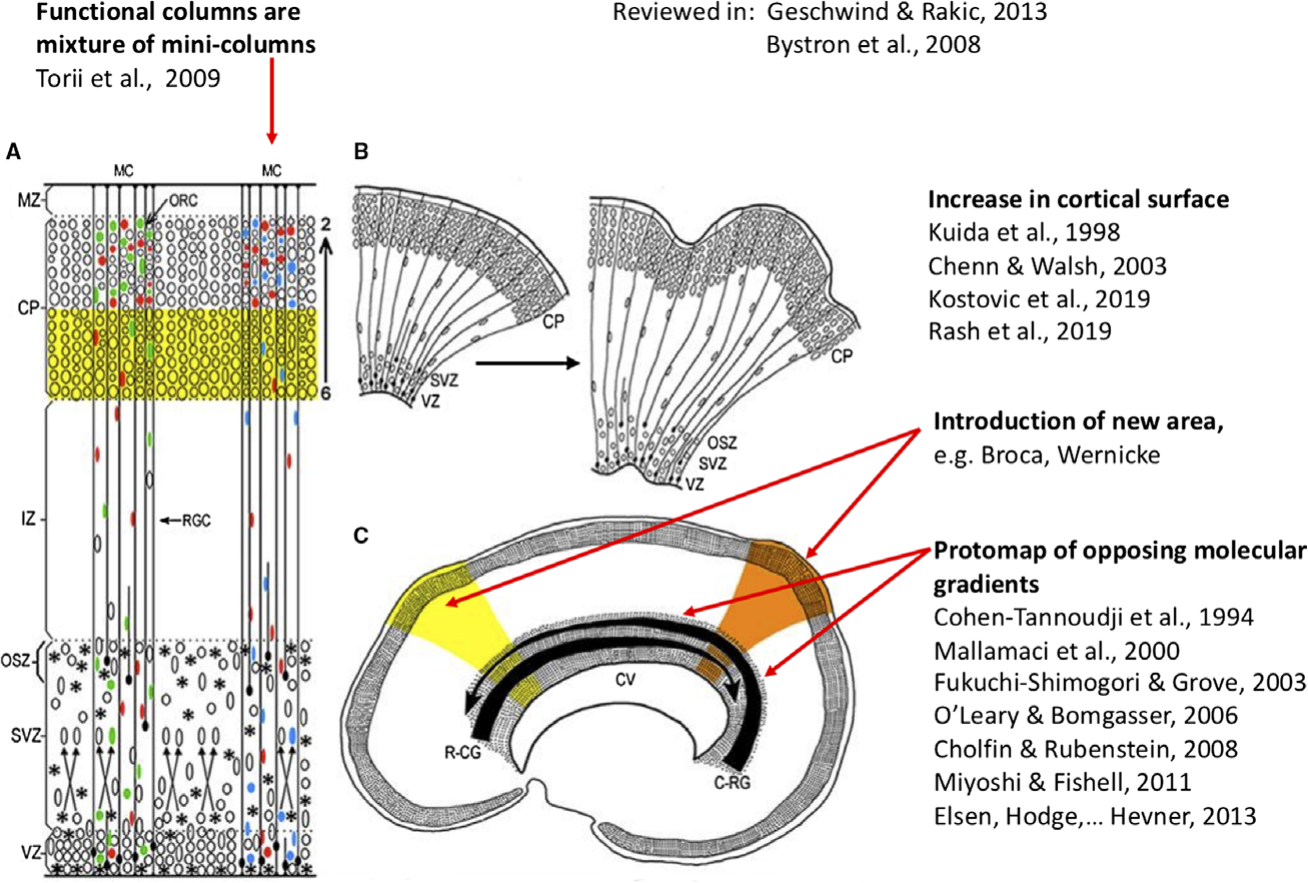
Sites of production and migration of neurons in the primate cerebral cortex. Diagrammatic presentation of the sites and pattern of radial migration in the developing cerebral cortex based on Torii et al. (2009), reviewed in Geschwind & Rakic (2013).

Characterization of radial glial cells (RGCs) at cellular, molecular and physiological levels is revealing considerable species‐specific differences (Rakic, 1988; Torii et al. 2009; Hansen et al. 2010; Nowakowski et al. 2017). Although rodent RGCs are an excellent model for the study of basic aspects of cortical development, their counterparts in primates, including humans, are considerably different with respect to their location, developmental potential and molecular signature. One of the earliest noted differences was that cytoskeletal proteins, such as glial fibrillary protein, that are essential to provide strength and durability to support the elongated curvilinear radial glial scaffold are enriched in the larger and more slowly developing human cerebrum (Rakic, 1988; Torii et al. 2009; Geschwind & Rakic, 2013). This concept has been supported by the use of more advanced methods that have also identified additional genetic and molecular differences (Lui et al. 2014). Additional subtypes of radial glia have been described in the developing human cortex, including outer (or basal) radial glia and a more recently described truncated form of radial glia, that differ from the previously described ventricular (or apical) radial glia in morphology, location, molecular identity and role in cortical development (Fietz et al. 2010; Hansen et al. 2010; Nowakowski et al. 2016, 2017). While outer radial glial cells (oRGCs) contribute neurons to all cortical layers, they appear to be the predominant source of upper cortical layer neurons in humans and other primates (Pollen et al. 2015; Nowakowski et al. 2016). Recent data show that after outer radial glia produce neurons for the superficial cortical layers, they generate various types of glial cells that eventually outnumber neurons and settle in all cortical layers as well as in the subcortical white matter where they contribute to regional differences in cerebral growth that contribute to the formation of convolutions (Duque et al. 2016; Rash et al. 2019). Evolutionary changes in basal progenitor morphology, in particular an increase in process numbers, is linked to its increased proliferative capacity in humans (Kalebic et al. 2019). Figure 1 illustrates the most recent data from non‐human primates that integrate the original idea of the radial model with revelations on the glial output of the outer subventricular zone (OSVZ).

## Role of intermediate progenitor cells in human corticogenesis

**Figure 2:**
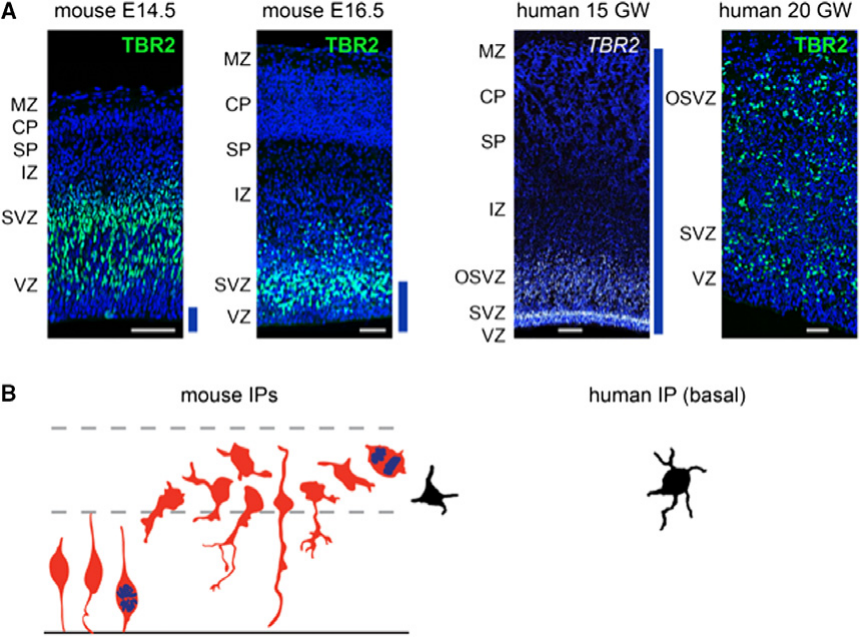
Intermediate progenitor cells (IPCs) in mice and humans. (A) TBR2 protein and mRNA are specifically expressed in IPCs. In mice, TBR2+ IPCs are located in the ventricular zone (VZ) and subventricular zone (SVZ); in humans, IPCs additionally occupy the outer (oO)SVZ, where they are generated from outer radial glial cells (oRGC). (Human 15 PCW *in situ* hybridization to detect TBR2 mRNA from Brainspan Atlas, Allen Institute.) Blue lines indicate cortical mantle thickness relative to human 15 GW. Scale bars: 50 μm for mouse E14.5, mouse E16.5 and human 20 GW; 250 μm for human 15 PCW. (B) Tracings of IPC morphologies from mouse and human cortex. Most IPCs in the mouse VZ are bipolar and contact the ventricle. In contrast, IPCs in the SVZ and (in humans) OSVZ have multipolar morphology. Human multipolar IPCs are reported to have more numerous processes than those in mice (Kalebic et al. 2019). IPC tracings in red are adapted from Mihalas & Hevner (2017); in black adapted from Kalebic et al. (2019).

Intermediate progenitor cells (IPCs) are a type of transit‐amplifying cell that produce small numbers of glutamatergic neurons, with average clone size ~2 neurons in mice (Vasistha et al. 2015; Mihalas & Hevner, 2017). In humans, IPCs are abundant in the oSVZ, where they are generated from oRGCs. As revealed by expression of TBR2 (an IPC‐specific marker), IPCs are located in the ventricular zone (VZ) and SVZ of mice, but are vastly expanded in the human developing cortex to also include the OSVZ, at much greater distances from the ventricle than in mice (Fig. 2A). Under regions of gyral growth, IPCs are even more numerous and occupy a thicker OSVZ than under developing sulci (Kriegstein et al. 2006). Thus, along with oRGCs, IPCs contribute to both radial expansion and gyrification of the human brain. Indeed, humans with abnormal IPCs due to deficient TBR2 expression have a severe cortical malformation, characterized by microcephaly (small brain size) and polymicrogyria, a defect of gyrus formation (Baala et al. 2007).

The morphology of IPCs is extremely complex, although less is known about this in humans than in mice (Fig. 2B). In the VZ, at least in mice, most IPCs exhibit short bipolar morphology, with their apical process attached to the ventricle (Kowalczyk et al. 2009; Hatakeyama et al. 2014). In the SVZ and OSVZ, IPCs usually exhibit multipolar morphology (Kowalczyk et al. 2009; Kalebic et al. 2019). The processes of IPCs exhibit dynamic extension and retraction, often contacting RGCs at the surface of the VZ. These processes express Dll1 protein, and thereby activate Notch signaling in RGCs at a distance (Nelson et al. 2013). Notch signaling is critical to prevent premature differentiation of radial glia, including oRG, in the OSVZ (Hansen et al. 2010). Recently, IPCs were reported to have more processes in humans as compared with mice (Kalebic et al. 2019). However, studies of human IPC morphology, gene expression and dynamics remain quite limited, and would be an interesting route for further investigations (Hevner, 2019).

## Specification of pyramidal neurons: layers, function, arealisation

**Figure 3:**
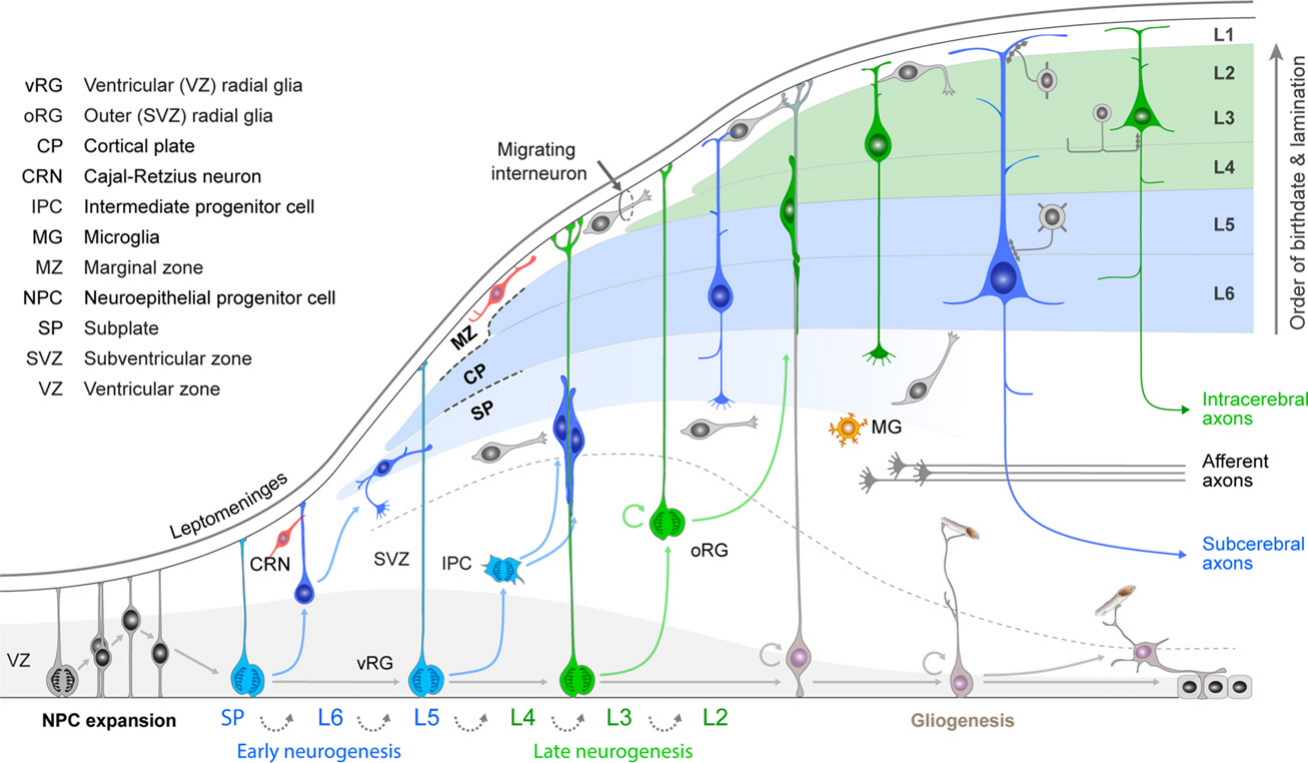
Schematic illustration of neurogenesis in the mammalian neocortex. Neuroepithelial cells (NPCs) undergo symmetric cell division to produce an initial pool of cortical progenitors that later transform into ventricular radial glia cells (vRGCs). vRGCs begin asymmetric cell division to generate another vRGC and a nascent projection neuron. The neuron then migrates radially from the ventricular zone (VZ) along the basal process of a RGC into the cortical plate (CP). The earliest born neurons migrate to form the preplate. Later migrating neurons split the preplate into the marginal zone (MZ) and subplate (SP). As neurogenesis proceeds, diverse subtypes of neurons are generated through the successive asymmetric division of RGCs. Early‐born nascent projection neurons settle in the deep layers (Layers 5 and 6; red layers), and later‐born projection neurons settle in towards mid‐neurogenesis stage. Additionally, some populations of RGC daughter cells become intermediate progenitor cells (IPCs) or outer radial glial cells (oRGCs) in the subventricular zone (SVZ). After the neurogenic stages, the radial scaffold detaches from the apical surface and vRGCs become gliogenic, generating astrocytes, or transform into ependymal cells. Tangential migration of interneurons is observed in the MZ, intermediate zone (IZ) and SVZ. Neocortical projection neurons mature into cortical projection neurons (CPNs), which show layer‐ and subtype‐specific morphology and axonal projection patterns. Adapted from Kwan et al. (2012).

The brain, unlike any other organ in our body, has a huge variety of different cell types that assemble into functional circuits that reflect a complex interaction between an unfolding genetic program and environmental interactions. The cerebral cortex is composed of a huge variety of distinct cell types that include neurons, glia and non‐epithelial tissue components (vasculature). The cerebral cortex shows considerable differences in the composition and proportions of these elements in both radial and tangential domains. The variations in cell composition and laminar proportions reflect the different computational functions these cortical areas have to perform (Brodmann, 1909), and emerge as development progresses. The sequential generation of discrete cell fates, and concerted migration to correct laminae, is critical for the assembly of the neocortex. After the generation of the preplate, the cortical plate neurons are generated in an inside first, outside last fashion (Rakic, 1988; Bystron et al. 2008; Fig. 3). The challenge is to understand how this remarkable heterogeneity of projection neuron types, interneurons and glial cells emerge from distinct neural stem cell lineages and assemble into circuits that show variation across different cortical areas. Recent lineage analysis experiments and mathematical modeling inquiries helped to increase our understanding how these processes are regulated (Beattie & Hippenmeyer, 2017; Picco et al. 2019).

Neuroepithelial stem cells (NPCs) produce all neural progenitor cells, with the duration and timing of progenitor cell division contributing both to the size of the resultant neocortex and the identities of its constituent cells (Picco et al. 2018). NPCs initially amplify their pool in fast cell cycle divisions, and enlarge the pool before they transform into ventricular radial glial progenitor cells (Fig. 2). Recent studies employing single‐cell analysis and clonal lineage tracing in various species suggest that neural stem cell and radial glial progenitor cell lineage progression are highly regulated to generate cell‐type diversity during cortical development. Mosaic analysis with double‐markers‐based quantitative clonal analysis suggests that the behavior of RGCs is remarkably coherent and predictable across all developmental stages (Hippenmeyer et al. 2013). RGCs in the neurogenic phase do not undergo terminal differentiation in a stochastic manner, but rather follow a defined program of cell cycle exit resulting in a unitary output of about 8–9 neurons per individual RGC. Resent research in mouse revealed that the size of asymmetric neurogenic clones is similar across neocortical areas, suggesting that the unitary neuronal output is a general property of cortical RGCs in mouse (Hippenmeyer et al. 2013).

An important aspect of RGC lineage is the areal identity that is conferred upon daughter cells. The protomap hypothesis states that the layout of the cerebral cortex is determined by the coordinated and compartmented expression of genes in time and space at the earliest stages of its development, prior to receiving sensory input (Rakic, 1988, 2009). Gene expression studies across the human lifespan show that divergent gene expression between cortical regions appears more evident at early fetal stages of development than peri‐ and postnatally, suggesting that marked inter‐areal transcriptional differences are important early in development (Pletikos et al. 2014; discussed in more detail below). Experiments in rodents have shown that certain transcription factors are expressed in gradients across the dorsal telencephalon, their expression controlled by gradients of soluble morphogens from discrete forebrain signaling centers and, in turn, transcription factors appear to control regional expression of cell adhesion molecules and cell surface receptors (Storm et al. 2006; O'Leary et al. 2007; Rakic et al. 2009; Alfano & Studer, 2013), leading to organization of area‐specific thalamocortical afferent (TCA) projections (López‐Bendito & Molnár, 2003; Zembrzycki et al. 2013). The question is whether such a developmental scheme is sufficient to produce all the extra areas of specialized association cortex, as well as the greatly increased interconnectivity between these areas, seen in primates (Buckner & Krienen, 2013). The evidence so far is that the human and mouse brain do share fundamental mechanisms of areal specification (Miller et al. 2014; Clowry et al. 2018); however, there are subtle differences that could lead us to a better understanding of cortical evolution and the origins of neurodevelopmental diseases. For instance, robust evidence has been found for reciprocal expression of COUP‐TFI and SP8 from anteromedial to posterolateral cortex (Fig. 3; Alzu'bi et al. 2017a), as has been observed in rodents (Borello et al. 2013). Rodent expression manipulation experiments show these transcription factors have mutually antagonistic effects; loss of COUP‐TFI function leads to expansion of frontal motor areas at the expense of posterior sensory areas (Armentano et al. 2007), whereas SP8 loss has the opposite effect (Sahara et al. 2007).

**Figure 4:**
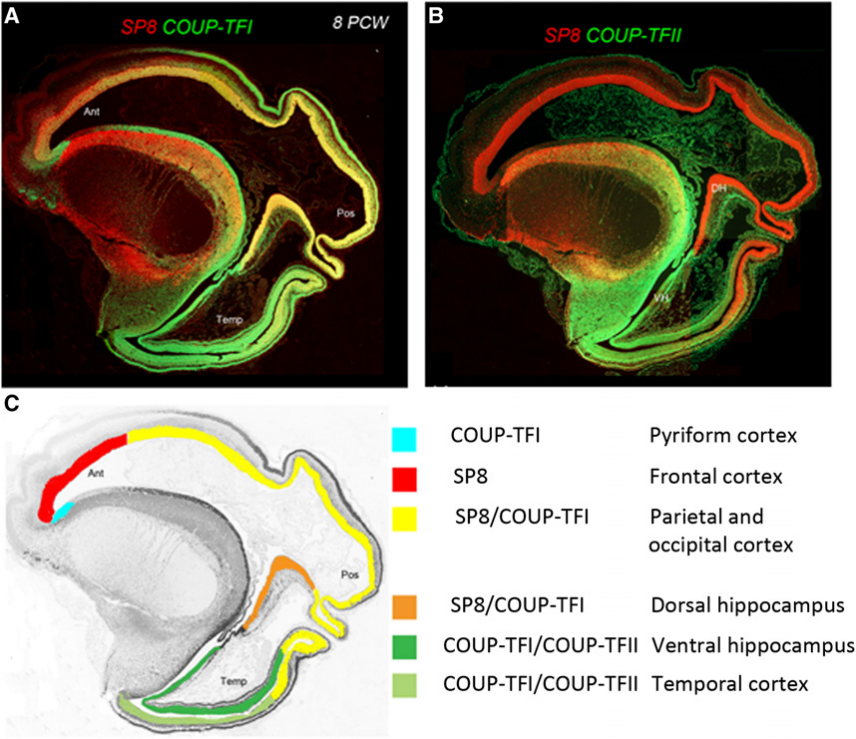
Protomap of the human cerebral cortex. Expression of opposing gradients of SP8 and COUP‐TFI in a sagittal section of human fetal telencephalon at 8 post‐conceptional weeks (PCW). (B) Compartmentalized expression of SP8 and COUP‐TFII in the developing cerebral cortex. (C) Summary of the findings in (A) and (B), demonstrating how the progenitor zones of the cortex are subdivided into compartments by combinatorial transcription factor expression that give rise to different functional areas of cortex in maturity (adapted from Alzu'bi et al. 2017a).

In humans, SP8 and COUP‐TFI overlap extensively in the VZ of visual, auditory and somatosensory cortex (Alzu'bi et al. 2017a), whereas in mouse COUP‐TFI and SP8 show little overlap (Borello et al. 2013). Combinatorial expression of COUP‐TFI and SP8 could maintain a common genetic identity for future primary sensory areas and a partially shared identity with SP8‐expressing frontal motor cortex with which these sensory areas will interconnect, along with allied association cortex, via dorsal sensorimotor pathways (Hickok & Poeppel, 2007; Milner & Goodale, 2008). In mouse, COUP‐TFII is confined to a very small portion of posterior cortex (Qiu et al. 1994) , but in human is expressed extensively throughout the ventral temporal and ventral posterior cortex overlapping with COUP‐TFI expression (Fig. 4; Alzu'bi et al. 2017a). Expansion of cortical COUP‐TFII expressing territory in human fetal brain may mirror the increased size and complexity of the association areas of the ventro‐temporal cortex including the ventral stream of cognitive visual processing (Milner & Goodale, 2008; Kaas, 2013).

An extension of this observation is that dorsal and ventral hippocampus are also differentiated by combinatorial expression of SP8/COUP‐TFI and COUP‐TFII/COUP‐TFI, respectively (Fig. 3; Alzu'bi et al. 2017a). Each domain has distinct functions and distinct afferent and efferent connections (Strange et al. 2014). In mouse, high to low expression of COUP‐TFI along a septo‐temporal gradient is important for the functional organization of the hippocampus (Flore et al. 2017). The protomap for human hippocampal specialization is laid down early in development, and determined by complementary expression of SP8 and COUP‐TFII rather than graded expression of COUP‐TFI.

Nevertheless, there is still a substantial role for input from TCAs in fine‐tuning the protomap during the neurogenic phase of cortical development (Dehay et al. 1996; Magrou et al. 2018), the significance of which has been heightened by the discovery of spontaneous activity in the thalamic nuclei prior to sensory innervation (Moreno‐Juan et al. 2017) and the finding that TCAs invade the cortical subplate much earlier in primates than in rodent models (Alzu'bi et al. 2019). TCAs may interact with the oSVZ, regulating mitogenesis (Dehay et al. 2001; Gerstmann et al. 2015), or with neurons in the SP re‐specifying their phenotype before they assume their final position in the lower layers of the cortex (Ozair et al. 2018). This should prove a rich area for research in the future.

## Interneuron generation and diversity, and their recruitment into the frontal association cortex

Unlike excitatory neurons, which are cortically derived, experiments in rodents have demonstrated that inhibitory interneurons are produced in the ganglionic eminences (GEs) and tangentially migrate into the cortex and, to a large extent, human fetal brain appears to share fundamental mechanisms for cortical interneuron development (Laclef & Métin, 2017). Landmark publications (Hansen et al. 2013; Ma et al. 2013) challenged the earlier notion that up to 65% of cortical interneurons may derive from the dorsal pallium (Letinic et al. 2002) and demonstrated a predominantly ventral pallial origin for interneurons. To allow for the greater abundance and diversity of cortical inhibitory interneurons (Ballesteros Yáñez et al. 2005; Povysheva et al. 2007; Raju et al. 2017), the human fetal brain has evolved an expanded oSVZ in the GE (Hansen et al. 2013); the caudal ganglionic eminence (CGE) has increased in complexity extending ventrally as the temporal lobe has increased in size (Hansen et al. 2013; Ma et al. 2013; Alzu'bi et al. 2017a) and contributes to relatively greater proportion (> 50%) of cortical interneurons (Hansen et al. 2013). The majority of interneuronal subtypes are characterized by the expression of parvalbumin (PV), somatostatin (SST) or calretinin (CalR). PV+ and SST+ interneurons are generated in the medial ganglionic eminence (MGE), whereas CalR+ interneurons are mainly generated in the CGE (Wonders & Anderson, 2006; Rudy et al. 2011). The ratio of PV+ and SST+ interneurons to projection neurons is similar in mouse and human, whereas CalR+ interneurons represent 13% of the total number of cortical neurons in human compared with only 4% in mouse (Hladnik et al. 2014). One half of all CalR+ interneurons in primates are in the frontal associative areas, representing almost 50% of all GABAergic interneurons in this region (Condé et al. 1994; Gabbott et al. 1997; Zaitsev et al. 2004; Barinka & Druga, 2010; Hladnik et al. 2014). Is the evolution of this extra contingent of CalR+ interneurons entirely dependent upon the evolution of the ventral CGE, or is it augmented by intracortical generation of interneurons?

**Figure 5:**
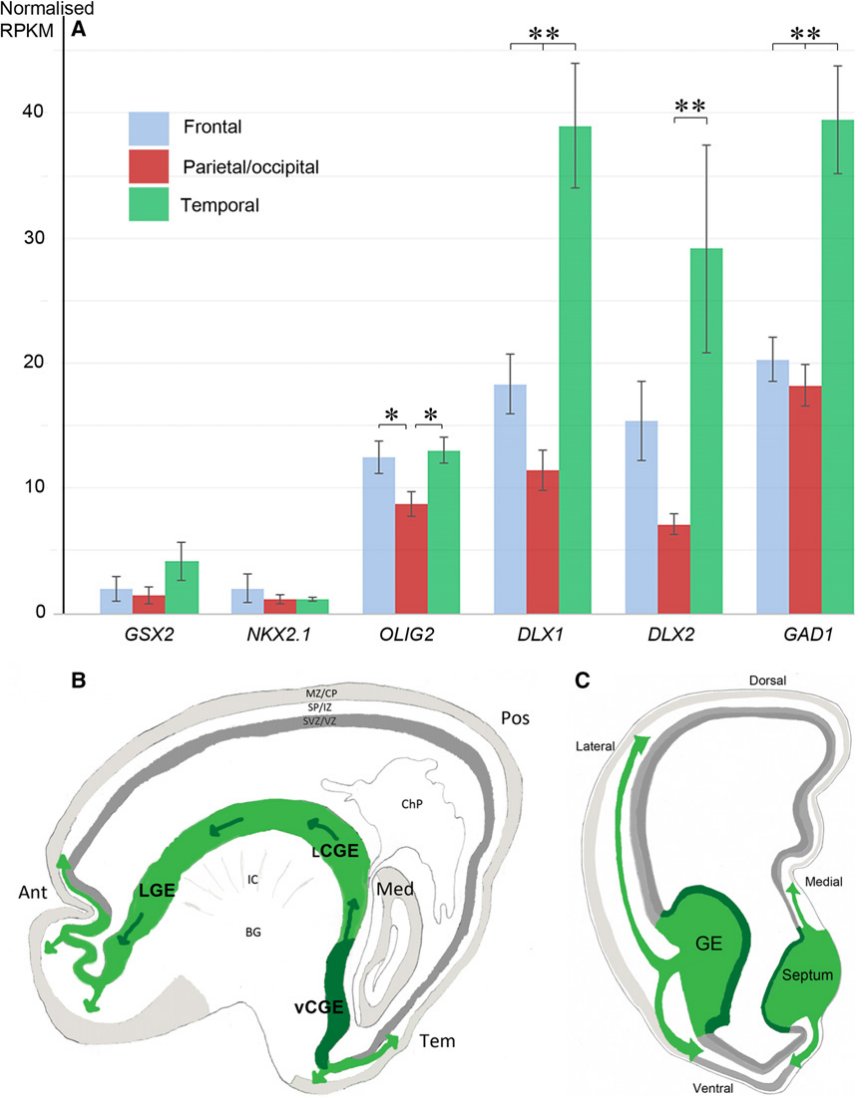
Generation of γ‐aminobutyric acid (GABA)ergic cortical interneurons in human. (A) Between 8 and 12 post‐conceptual weeks (PCW) expression of early ‘GABAergic’ genes associated with ventral telencephalic domains in rodents is also very low in the human dorsal telencephon, but ‘GABAergic’ genes expressed in late progenitor and post‐mitotic cells shower higher expression, particularly in frontal temporal cortex. (B) and (C) Interneuron migration pathways proposed to be more prominent in human than mouse, including an anterior pathway from caudal ganglionic eminence (CGE) to frontal cortex and a medial pathway from septum to frontomedial cortex.

In cases of human holoprosencephaly with severe hypoplasia in ventral telencephalon, interneurons of MGE origin were absent or severely reduced, whereas CalR+ interneurons are present in the cortex of these cases (Fertuzinhos et al. 2009), suggesting the presence of an additional source of CalR‐expressing interneurons other than the GEs. The neuronal progenitor marker GSX2, proposed to specify CalR+ interneurons generation in the CGE, has been found to be expressed in proliferating cells in the VZ/SVZ of the human fetal cortex (Radonjić et al. 2014). Cultures differentiated from isolated human cortical progenitors from anterior and posterior cortex were enriched with GABA+ and CalR+ cells, and the markers of CGE‐derived cells COUP‐TFI and COUP‐TFII, even though these cultures lacked expression of MGE cell marker NKX2.1. Given the high levels of CalR expressed by cultured GABA+ cells, it would seem that if cortical interneurogenesis exists it is primarily to contribute to CGE‐like (CalR+) interneuron populations (Alzu'bi et al. 2017b). COUP‐TFII, CalR and GABA expressing cells were present in higher proportions in anterior compared with posterior cortex‐derived cultures, suggesting potential regional variation in generation of interneurons in the dorsal telencephalon (Alzu'bi et al. 2017b). Similarly, the mRNA levels (RNA‐seq analysis) for several genes involved in the genetic regulatory pathway of interneuron specification (Fig. 5A) tended to be more highly expressed in samples derived from anterior than posterior cortical regions of early human fetal cortex (Lindsay et al. 2016; http://www.hdbr.org/expression/); which is also consistent with *in situ* hybridization and quantitative polymerase chain reaction (qPCR) studies reporting elevated expression of various ‘GABAergic’ genes anteriorly, including *CALB2* (CalR; Bayatti et al. 2008; Ip et al. 2010; Al‐Jaberi et al. 2015). Furthermore, a single‐cell RNA‐seq study of early developing human prefrontal cortex also found interneuron progenitors, although they may not have entered the cell cycle (Zhong et al. 2018). Collectively, these data suggest that the anterior (frontal) cortex could be a preferred region for generating fractions of GABAergic interneurons in human fetal brain.

However, the highest expression of these genes was generally seen in the temporal lobe (Fig. 5A). Therefore, an alternative explanation for elevated ‘GABAergic’ gene expression in the frontal and temporal cortex of human fetal brain and the high proportion of CalR+ interneurons in the prefrontal cortex of adult human brain could be that migrating interneurons, and maybe mitotic progenitors, more rapidly invade frontal and temporal regions than the posterior cortex, even from apparently caudal subpallial structures such as the CGE (Alzu'bi et al. 2017b; Clowry et al. 2018). CGE‐derived interneurons follow multiple migratory streams to reach their target regions, which appear to be temporally and spatially distinct, identified by expression of the characteristic transcription factors COUP‐TFI, COUP‐TFII and SP8 (Alzu'bi et al. 2017a,2017b). In addition to the caudal migratory stream directing CGE‐derived cells into the temporal cortex and hippocampus (Yozu et al. 2005), these cells can also migrate anteriorly via the LGE into the anterior and lateral cortical regions (Fig. 5; Touzot et al. 2016; Alzu'bi et al. 2017b), which could also explain the higher populations of CalR+ interneurons in the frontal regions compared with other cortical areas in the developing fetal brain of human and monkey (Ma et al. 2013). Such a pathway cannot be proposed as evolutionarily novel to the human as it has also been reported in mouse (Touzot et al. 2016), but it might be more important in human as it facilitates CalR+ interneurons reaching the expanded and evolved frontal cortex (Hladnik et al. 2014). The newly described medial migratory route of interneurons from the subpallial septum into the medial wall of the anterior cortical regions, missing or overlooked in rodents (Rubin et al. 2010), could also contribute to the increasing GABAergic gene expression in the frontal cortex of human fetal brain (Fig. 5C). DLX2+, OLIG2+ and GAD67+ cells from the septum can enter the dorsal and ventral medial walls of the anterior cortical regions, some of which retain the potential to divide (Alzu'bi et al. 2017b; Alzu'bi & Clowry, 2019).

Finally, an extended period of interneuronal generation and migration from the GE into the prefrontal cortex, in particular, has been reported in human (Arshad et al. 2015; Paredes et al. 2016). Interneuron progenitors (proliferating DLX2+ cells) in CGE, coinciding with interneuron migration into the cortex, were abundant beyond 35 post‐conceptual weeks (PCW) in human, indicating that cortical interneuron generation in this domain persists until late stages of human pregnancy (Arshad et al. 2015). In addition, the presence of a migratory stream of large numbers of doublecortin+/GABA+ young neurons has been reported (Paredes et al. 2016) that form a cap‐like structure surrounding the anterior body of the lateral ventricle, and continue to migrate and integrate into the human frontal cortex 5 months after birth. These cells express transcription factors associated with both MGE‐ (NKX2.1 and LHX6) and CGE‐ (SP8 and COUP‐TFII) derived interneurons (Paredes et al. 2016), and represent various interneuronal subtypes that express neuropeptide Y, SST, CalR, calbindin and secretagogin (Paredes et al. 2016; Raju et al. 2017). As the initiation of the critical period of plasticity is directly linked to the maturation of inhibitory circuitry (Hensch, 2005; Gandhi et al. 2008; Southwell et al. 2010), this late integration of interneurons into the human frontal cortex could extend the period of plasticity in this region (Paredes et al. 2016).

Many neurodevelopmental conditions, such as autism spectrum disorders and schizophrenia, are associated with an imbalance of excitatory and inhibitory neurons in the prefrontal cortex (Bicks et al. 2015; Canitano & Pallagrosi, 2017). Therefore, a complete understanding of their origin will be required to completely understand the etiology of these syndromes.

## The importance of the subplate to human cortical development

**Figure 6:**
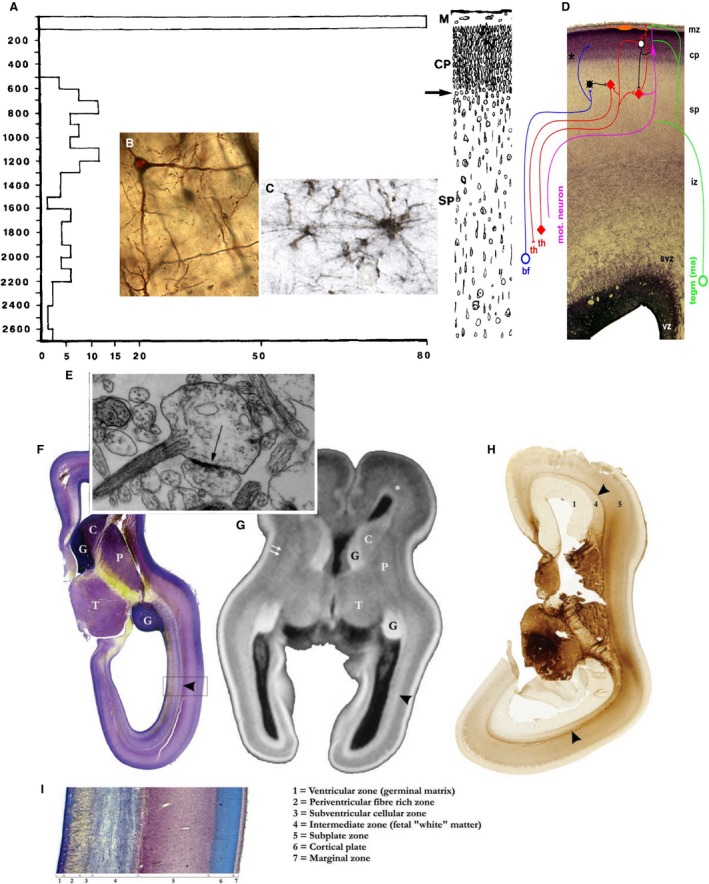
The human subplate. Summary figure showing synaptic distribution (A), neurons (B), early astroglia (C), transient circuitry (D), synapse (E) and delineation of subplate (F–I). Graphic representation of the spatial distribution of synapses (A) in the somatosensory cortex of a 15‐week‐old human fetus obtained in 23 probes each 5 μm wide, in the abscissa as a function of 100 μm (with permission from Kostović & Rakic, 1990). Golgi impregnated large neuron (B) with long smooth dendrites (with permission from Kostović et al. 2019). ‘Transient’ astroglia in the subplate (C) (with permission from Kostović et al. 2019). Electromicrograph (E) of the growth‐cone‐like dendritic profile forming a synaptic junction (arrow) in the subplate of 21‐week‐old human fetus (with permission from Kostović & Rakic, 1990). Transient circuitry of subplate (D) in a 24‐week human fetus consists of thalamus‐subplate‐thalamus circuit (red), basal forebrain (blue) and monoaminergic input (green), connections between SP neurons and transient projection to the cortical plate (with permission from Kostović & Judaš, 2007). Comparative images of subplate on Nissl (F, I), MR *in vitro* (G) and AChE preparations (H) show that subplate is the thickest compartment of the cerebral wall situated between cortical plate and external sagittal stratum (arrowhead) (with permission from Kostović et al. 2002).

The subplate is the most voluminous, transient compartment in the fetal cerebral wall composed of post‐migratory and migratory neurons, glia and growing axonal plexi, which are embedded in abundant extracellular matrix (Kostović & Rakic, 1990; Hoerder‐Suabedissen & Molnár, 2015; Fig. 6). The crucial role of the subplate in development of cortical connectivity and other aspects of corticogenesis was recognized across various disciplines: in‐growth of thalamocortical and basal forebrain afferents (Kostović & Rakic, 1990; Allendoerfer & Shatz, 1994; Hoerder‐Suabedissen & Molnár, 2015; Alzu'bi et al. 2019), neuronal differentiation (Kostović et al. 2019), gliogenesis (Kostović et al. 2019), molecular specification of neurons (Bayatti et al. 2008; Hoerder‐Suabedissen & Molnár, 2015), development of columnar organization and microcircuitry (Allendoerfer & Shatz, 1994), spontaneous activity (Kanold & Luhmann, 2010), evoked activity (Allendoerfer & Shatz, 1994), neurosecretion (Kondo et al. 2015; Adorján et al. 2019), and specification of migratory neurons (Ohtaka‐Maruyama et al. 2018; Ozair et al. 2018). The interest in the human subplate increased after successful visualization of subplate *in vitro* and *in vivo* magnetic resonance imaging (MRI) and evidence of vulnerability in hypoxia/ischemia (Sheikh et al. 2018). The functional activity of the subplate in human fetus remains largely unexplored (Kostović & Judaš, 2007). Accumulating evidence for the essential role of the subplate in development of large‐scale connectivity, microcircuitry and synaptogenesis in human fetal brain led to the view that disturbance of subplate may be a pathogenic factor in autism, schizophrenia and other circuitry disorders (Hoerder‐Suabedissen & Molnár, 2015; Kostović et al. 2019). Despite numerous studies, the subplate is still considered an enigmatic zone.

There are several questions to be answered that will require new experimental approaches. The first is the problem of production of an extraordinary amount of extracellular matrix. It was shown that laminar shifts of extracellular matrix closely follow all developmental phases of subplate (Kostović et al. 2019). Second, what are the unique features in the process of expansion of subplate in primates as discussed by Kostović et al. (2019)? Thirdly, how does the understanding of the early differentiation of glia (Kostović et al. 2019) and neurosecretory functions (Kondo et al. 2015; Adorján et al. 2019) shed new light on the complexity of subplate? There is general agreement that subplate neurons are among the earliest born neurons in the cerebral cortex (Duque et al. 2016). However, there is less agreement about their site of origin (Hoerder‐Suabedissen & Molnár, 2015), and their interactions with migratory neurons (Ohtaka‐Maruyama et al. 2018; Ozair et al. 2018). Finally, what drives the secondary expansion of subplate (Duque et al. 2016) and what is the fate of subplate neurons (Sedmak & Judaš, 2019)?

It is important to note that in humans the subplate exists from 8 to 42 weeks of gestation, and passes through many phases of structural‐functional organization. In general, the neurogenesis, structural and molecular differentiation, gene expression and pathway content of the subplate are better known than its functional activity. Early spontaneous activity seen in the subplate in animal models has been attributed to non‐synaptic intercellular junctions (Kanold & Luhmann, 2010), and is also observed in the human subplate (Moore et al. 2011). Between 15 and 25 weeks, synapses are increasing in the subplate, as observed by electron microscopy (Kostović & Rakic, 1990), and synaptic activity may become the mechanism for conveying input from afferent axons to subplate (Allendoerfer & Shatz, 1994; Kostović & Judaš, 2007). However, at 19–21 PCW, *in vitro* electrophysiological studies have suggested that functioning synaptic contacts are rare (Moore et al. 2011). After 24 PCW when the first synapses develop in the cortical plate, there is a gradual shift in activity towards cortical plate circuitry with parallel changes in (spontaneous) electroencephalogram (EEG) and evoked potentials (Kostović & Judaš, 2007; Kanold & Luhmann, 2010). However, it is very likely that subplate circuitry participates in these electrical phenomena, even after the beginning of cortical plate synaptogenesis. It is also proposed that activity from the subplate circuitry is propagated to other CNS regions, including the spinal cord (Hadders‐Algra, 2018), as well as participating in resting state activity and inter‐regional connectivity in the cortex. Confirming these hypotheses and elucidating the role it plays in development should be the subject of future studies. The diversity of communication mechanisms within the subplate compartment via transient inputs, ECM, glia and neurosecretion represent unexplored functional and morphogenetic features of the subplate in the large human gyrencephalic brain. These unexplored issues are prospective milestones for the study of disorders characterized by abnormal circuitry. Interestingly, the subplate layer and abnormal processes therein, or impact of disease on it are all becoming increasing tractable in developing humans through high‐resolution MRI of both living infants and fixed brains.

## Gene expression networks and neurodevelopmental disease genes

Human brain development is characterized by large‐scale changes in cellular composition and cytoarchitecture, during which various neurodevelopmental processes such as neurogenesis, synaptogenesis and myelination progress with distinct trajectories and over a prolonged period of time (Silbereis et al. 2016). Over the last decade, several important genomic resources have been generated to facilitate the study of the human brain, with prominent examples including Kang et al. (2011), Hawrylycz et al. (2012), Miller et al. (2014), Fromer et al. (2016), GTEx consortium (2015) Won et al. (2016), Lake et al. (2016) and Li et al. (2018). Unfortunately, few studies attempted integrative multi‐dimensional genomic analyses across developmental timepoints, and at the levels of different brain regions, cell types and single cells.

To capture the transcriptional signatures of the developmental processes, early microarray‐based bulk tissue studies have profiled gene expression with relatively extensive sampling across brain regions and/or timepoints (Johnson et al. 2009; Colantuoni et al. 2011; Kang et al. 2011). The Kang et al. (2011) study is notable for its comprehensive combination of spatial and temporal coverage that included 15 prenatal and postnatal periods. This study found that over 80% of protein‐coding genes are expressed in neocortical cells and change expression over time (Kang et al. 2011). Spatiotemporal expression patterns vary across genes and reflect the timing of known neurodevelopmental events, such as neurogenesis, myelination and synaptogenesis. The greatest variation between samples appears to correspond to the developmental period of the sample, with prenatal periods exhibiting the most extensive transcriptional changes between periods (Colantuoni et al. 2011; Kang et al. 2011; Jaffe et al. 2015). Likewise, transcriptional differences between neocortical areas are more pronounced during early to mid‐fetal periods than during postnatal periods. The number of differentially expressed genes between neocortical areas drops substantially in late fetal and early postnatal periods, and then rises to some degree in later periods (Pletikos et al. 2014; Li et al. 2018). The relatively large degree of spatial differential expression in prenatal neocortex may in part reflect the processes involved in the establishment of areal identity occurring at those stages.

Because bulk genomic analyses aggregate data across the diverse range of cell types found in the brain, it is essential to analyze gene expression within individual cell populations. Thus, the ability to determine gene expression pattern of a small number of cells or single cells is of great importance for resolving a variety of problems in the context of human brain development. Recent advances in single‐cell genomics offer the ability to characterize cell‐type diversity in a massively parallel and hopefully unbiased fashion. An increasing number of cell types and subtypes in the developing and adult human cerebral cortex have been characterized in a systematic manner at an unprecedented pace using single‐cell or nucleus RNA‐seq (Florio et al. 2015; Pollen et al. 2015; Lake et al. 2016; Onorati et al. 2016; Nowakowski et al. 2017; Fan et al. 2018; Li et al. 2018; Zhong et al. 2018; Mayer et al. 2019). These studies not only confirmed the transcriptomic signatures and maturational states of well‐established cell types, but also discovered previously unknown gene expression patterns in distinct cell types in the human brain. With increasing reproducibility and scalability, single‐cell genomic technologies undoubtedly have become and will remain important experimental platforms for cell‐type discovery in the developing human cortex.

Recent advances in genetics and genomics have identified hundreds of genetic coding and non‐coding variants that increase the risk for major neuropsychiatric disorders, including autism spectrum disorder (ASD; Šestan & State, 2018; Sullivan & Geschwind, 2019). While these advances have improved our understanding of the genetic architecture of neuropsychiatric disorders, identifying the molecular and cellular mechanisms underlying these disorders remains problematic because of the heterogeneity of risk loci, their varied protein functions, and the involvement of multiple cell types and brain regions. Consequently, information regarding gene regulatory and protein–protein interaction networks, as well as the distribution of these networks across the spatiotemporal dimensions of the human brain, is essential for clarifying when, where and what cell types are relevant to the etiology and treatment of ASD and other related disorders. Gene co‐expression analyses have also revealed that the developing human brain transcriptome can be organized into distinct co‐expression networks with often prominent spatial (cell type and brain region) or temporal patterns, and enriched for specific biological functions (Johnson et al. 2009; Kang et al. 2011; Miller et al. 2014; Pletikos et al. 2014). Interestingly, genetic variations in some of the most well‐connected genes in the networks with dynamic patterns have previously been linked to psychiatric or neurological disorders, including ASD and schizophrenia, suggesting that among disparate risk genes they may have converging functions in specific brain regions, cell types, circuits and developmental periods (Gulsuner et al. 2013; Parikshak et al. 2013; Willsey et al. 2013; Grove et al. 2019). Moreover, these networks can help identify the biological pathways that contribute to pathophysiology and implicate additional disease risk genes with shared co‐expression patterns.

**Figure 7:**
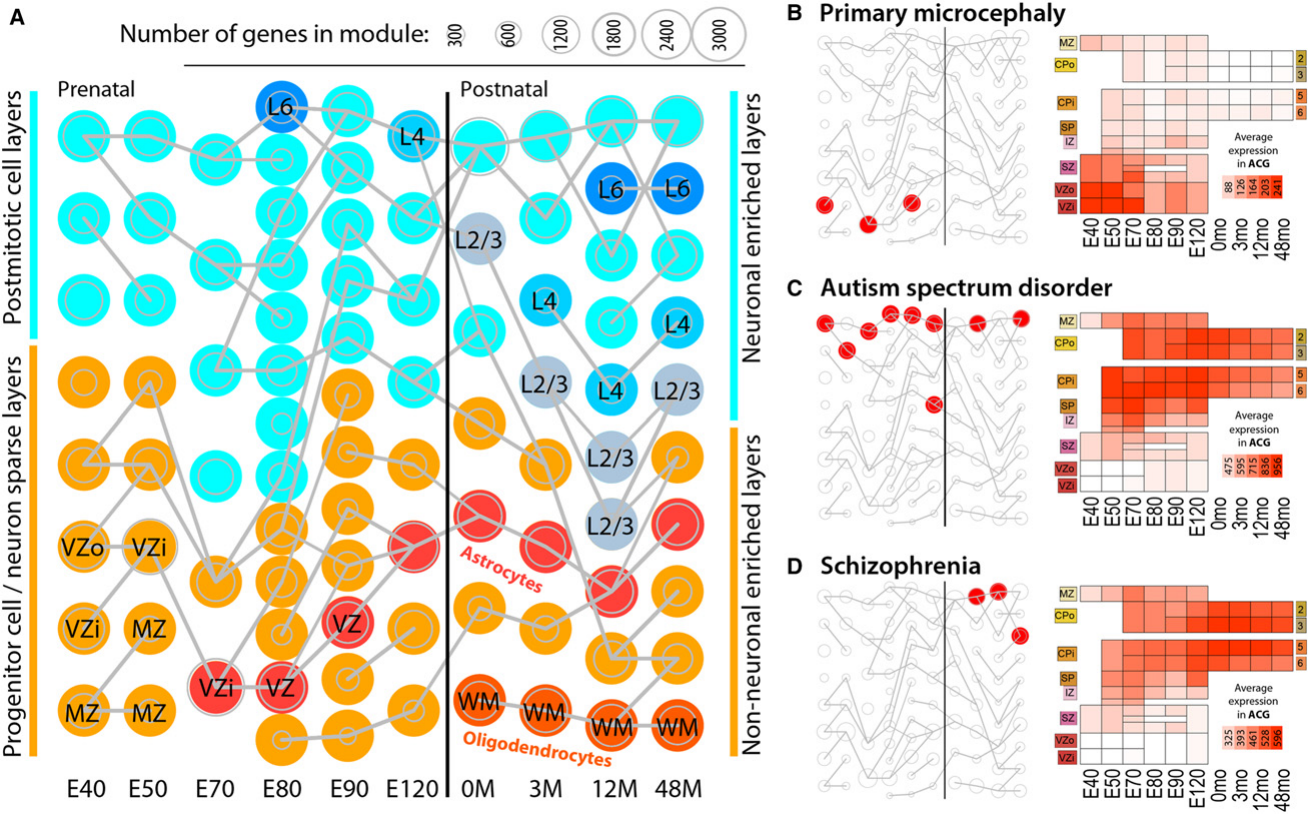
Spatiotemporal localization of disease‐associated genes in cortical cell populations during non‐human primate brain development. (A) Weighted correlation network analysis (WGCNA) co‐expression networks (gene modules) based on 603 laser‐microdissected samples from layers of primary visual and anterior cingulate cortex from rhesus monkey brain. WGCNA was run independently at each age (columns), including six prenatal timepoints (reported in days post‐conception) spanning early to mid‐fetal development, and four postnatal timepoints from birth to young adulthood. Modules were tested for significant gene set enrichment and overlap with hypergeometric tests. Modules significantly enriched for markers of glial cell classes (*P* < 10^−15^) or cortical layers (*P* < 10^−30^) are color‐coded and annotated. The remaining modules are colored or labeled based on maximal expression in post‐mitotic (neuron‐enriched; cyan) layers or progenitor or largely non‐neuronal (WM, layer 1; orange) layers. Modules from adjacent ages with the most highly significant gene overlap (*P* < 10^−50^) are connected by gray lines. (B–D) Left: modules significantly enriched for risk genes associated with neurodevelopmental disorders (empirically corrected *P* < 0.1; red discs). Right: average expression pattern of genes found in at least two enriched modules. Heat maps are organized by dissected layer and age. MZ, marginal zone; CPo/CPi, outer/inner cortical plate; SP, subplate; IZ, intermediate zone; SZ, subventricular zone; VZo/VZi, outer/inner ventricular zone. (B) Genes related to primary autosomal recessive microcephaly are enriched in early non‐neuronal modules, and show maximal expression in prenatal ventricular zone. (C) Genes related to autism spectrum disorder (ASD) are enriched in modules associated with cortical neurons and show highest expression in cortical plate across development. (D) Genes related to schizophrenia show similar neuronal layer enrichment to autism genes, but restricted to postnatal ages. The figure is reproduced from Bakken et al. (2016).

Cellular resolution expression profiling can differentiate between expression changes within cell types and changes in the frequencies of distinct cell types. As a step in this direction, expression levels of small populations of cells in individual cortical layers were profiled in rhesus monkey brain at 10 ages using laser‐capture micro‐dissection (Bakken et al. 2016; Fig. 7). This study found prolonged maturation of neurons through young adulthood with large changes in layer markers over development. Risk genes associated with ASD and schizophrenia were significantly enriched among genes co‐expressed in post‐mitotic cortical neurons, while genes associated with primary microcephaly were restricted to neuronal progenitor cells. More recently, single‐cell RNA‐sequencing of thousands of cortical cells in mid‐gestation human brain (Nowakowski et al. 2017) identified gene networks that contributed to cell differentiation, maturation and areal origin. One network included genes regulating the mTOR signaling pathway and showed enriched expression in outer radial glia. Moreover, the mTOR pathway enrichment of outer radial glia (basal, truncated) appears to be a human‐specific feature as it is not observed in developing non‐human primate cortex (Pollen et al. 2019). Thus, this progenitor cell population may be selectively vulnerable to genetic mutations in this pathway. It is therefore not surprising that a broad spectrum of malformations of cortical development, such as focal cortical dysplasia and tuberous sclerosis complex, have been linked to either germline or somatic mutations in mTOR pathway‐related genes (Mühlebner et al. 2019).

Of course, transcriptomic data do not always equate directly to protein expression (Mertins et al. 2016; Zhang et al. 2016), and do not capture information about post‐translational modifications of proteins. In order to address these problems, proteomic studies of the developing human brain are beginning to appear. Mass spectroscopy can be applied to formalin‐fixed paraffin‐embedded brain samples allowing quantification of protein expression (Deeb et al. 2015) and recently Djuric et al. (2017) were able to demonstrate time‐specific protein abundance changes between proliferative and post‐mitotic layers of the cerebral cortex, and compare these with proteomic data derived from human induced pluripotent stem cell‐derived neural precursors induced to differentiate under defined culture conditions.

Furthermore, in neurons in particular, proteins may be localized to cellular compartments far removed from the cell body, for instance on axon terminals or dendrites, and such information is not provided by single‐cell RNA‐seq or *in situ* hybridization studies. For instance, Fietz et al. (2010), using anti‐phosphovimentin immunohistochemistry, demonstrated that human outer radial glia retain a basal process during the M phase of cell division. Harkin et al. (2017) confirmed transcriptomic data demonstrating expression of neurexins in the developing cortical wall. Neurexins 1 and 3 showed co‐localized expression, principally to cell bodies in the cortical plate but also in proliferative zones, whereas Neurexin 2α was localized to growing axons of the intermediate zone and subplate.

A host of new molecular tools will provide additional insights into the molecular programs that contribute to human cortical development. High‐resolution maps of chromosome interactions can reveal the functional significance of disease‐associated mutations in non‐coding regions of the genome. For example, Hi‐C profiling of progenitor and post‐mitotic cell populations from fetal human cortex identified genes that are likely regulated by genetic risk variants for schizophrenia (Won et al. 2016). Spatial transcriptomics methods enable *in situ* molecular profiling of cells in their circuit context. For example, osmFISH revealed the spatial distributions of 31 cell types in adult mouse cortex (Codeluppi et al. 2018), and this technique among others can be applied to human cortex during development. High‐throughput, multi‐modal characterization of human cortical cells can identify the functional consequences of transcriptional profiles, including responsiveness to neurotransmitter signaling (Mayer et al. 2019). Cellular profiling in model systems can complement work in human tissue. Cell lineage has been inferred from gene expression trajectories, and has been directly measured in zebrafish brain by tagging cells with genetic barcodes using CRISPR‐Cas9 (Raj et al. 2018). This technology can be used in mammalian model organisms to capture a much more detailed picture of cortical cell fate decisions. Finally, human cerebral organoids are emerging as a powerful platform to begin to define causal roles for the growing list of genetic mutations associated with neuropsychiatric diseases.

## Organoids recapitulate *in vivo* development

Currently there is a vast gap in our knowledge regarding early human brain development, as well as neuro‐psychiatric disorders and their etiology. Studying human brain development used to rely on descriptive histological studies of post mortem specimens, and our ability to ask mechanistic questions was rather limited (Clowry et al. 2010; Molnár, 2011). Organoids, in < 10 years, changed all this. Organoid models for kidney, liver interstitial tissue, brain and eye formation have been developed and are now widely used to study early human development. However, current brain organoid models have acknowledged limitations. They exploit Matrigel, an ill‐defined and poorly controlled substrate matrix, to support organoid growth that requires more advanced 3D spatial control and stimulation. The media used for these studies are not optimal to investigate metabolic factors (for review, see Gilbert‐Jaramillo et al. 2019). The organoids currently lack vasculature, limiting our ability to investigate processes that rely on neurovascular interactions or barrier functions. The implementation of culturing protocols that introduce spatially organized and polarized organoid models will have a great effect on human developmental studies (Arlotta & Pasca, 2019). There is a great need for interactions with bioengineers to generate 3D culturing protocols that create spatially organized brain organoids, and include morphogen gradients that establish a dorso‐ventral and anterior‐posterior axis (Armstrong & Stevens, 2019), although the most advanced techniques currently in use already result in partial replication of radially organized units equivalent to the layered cortical plate, and chondroitin proteoglycan sulfide staining has even suggested the presence of a rudimentary, subplate‐like compartment in some organoids (Lancaster et al. 2017).

However, the utility of organoids for translational science has been proved following the recent outbreak of microcephaly related to ZIKV infection experienced throughout the world, but particularly in Brazil (Barbeito‐Andrés et al. 2018). In response to this health emergency, brain organoids engineered to mimic the developing human cerebral cortex were employed to model ZIKV‐induced microcephaly. Compared with monolayer or neurosphere cultures, brain organoids better recapitulate the composition, diversity and organization of cell types found in the developing cortex. By exposing the whole organoid to ZIKV, it was possible to identify which cortical cell types were efficiently infected by ZIKV. Studying forebrain organoids transiently exposed to virus at different developmental stages showed that ZIKV preferentially infected ventricular RGCs and oRGCs, over intermediate neural progenitors or immature neurons (Qian et al. 2016). The ZIKV tropism for neural progenitors was also observed *in vitro* with fetal human brain slices (Onorati et al. 2016;  Retallack et al. 2016), and *in vivo* by injecting ZIKV intraperitoneally into pregnant mice (Wu et al. 2016) or directly into the lateral ventricles of embryonic mouse brains (Li et al. 2016; Nguyen et al. 2016). Infected RGCs produced more infectious viral particles, causing an increased number of infected cells over time (Qian et al. 2016). Interestingly, ZIKV infection‐induced NPC death proved to be much more pronounced in organoids than in monolayer cultures (Garcez et al. 2016; Qian et al. 2016; Tang et al. 2016). ZIKV infection depletes RGCs by suppression of proliferation, and there is increased cell death of both infected RGCs and uninfected neurons causing a decrease in volume of both proliferative and post‐mitotic layers, generating microcephaly. In addition to providing the first biological link to the causality between ZIKV and microcephaly and insights on how ZIKV impacts brain development, human brain organoids have also permitted the screening of potential pharmacological treatments to mitigate ZIKV infection (Watanabe et al. 2018).

## Imaging the origins of neurodevelopmental disability

Modern tools of neuroimaging are now routinely used to reduce the incidence and severity of neurological impairment caused by problems around the time of birth. Bench‐to‐bedside imaging research strategy is applied to investigate the mechanisms of perinatal brain injury in order to find effective therapies, and have developed the first successful treatment for birth asphyxia – hypothermic neural rescue therapy (Azzopardi et al. 2014). Using bioinformatics methods to correlate histopathological observations with clinical imaging and genetic analysis is making a huge impact on our understanding of human brain development and damage (Edwards et al. 2018).

**Figure 8:**
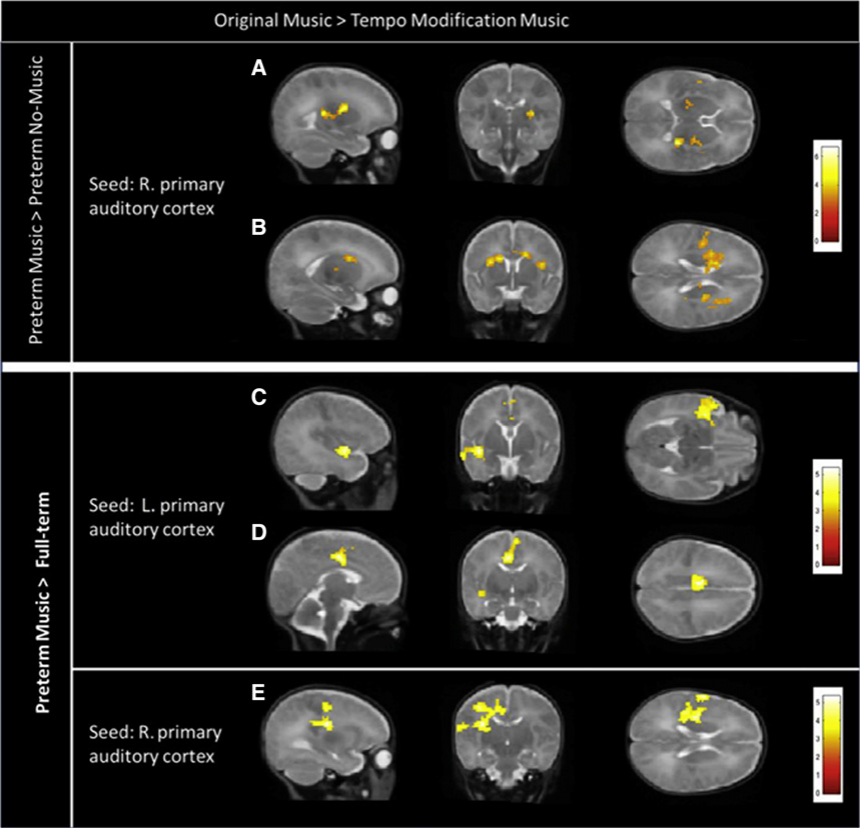
Impact of neurosensory stimuli on cortical development. Neonatal fMRI reveals enhanced music processing in preterm and full‐term newborns exposed to music starting from 33 weeks post‐conceptional age until term equivalent age compared with two additional groups without music intervention; preterm infants and full‐term newborns. Each row shows in a sagittal, coronal, and axial plane, results of the PPI analysis for Original  > Tempo‐Modification conditions (*P* < 0.05 FWE at cluster level). (A, B) Enhanced connectivity in Preterm‐Music compared with Preterm‐Control group between right primary auditory cortex (seed) and (A) the right thalamus and (B) the left caudate nucleus and middle cingulate cortex (MCC;* P* < 0.01 FWE at cluster level). (C, D) Enhanced connectivity in Preterm‐Music compared with Full‐Term group between left primary auditory cortex (seed) and (C) the left superior temporal gyrus and (D) the MCC. (E) Enhanced connectivity in Preterm‐Music compared with Full‐Term group between right primary auditory cortex (seed) and (E) the left MCC cortex and left putamen. Figure adopted from Lordier et al. (2019a) NeuroImage with permission from Elsevier.

Early life events such as premature birth and low birth weight due to poor fetal environment are important modulators of brain development with neurological and neuropsychiatric consequences through childhood and into adult life. Seven–12% of all births worldwide occur prematurely, 1% of all infants are born very premature and 35% of them develop neurodevelopmental impairments. Evaluating longitudinal cohorts of preterm infants that are followed‐up both with newly developed neuroimaging tools combined with motor, cognitive and behavioral evaluations have in recent years provided new knowledge on how early brain development is affected by these early life events. These studies have allowed us to identify altered global brain tissue growth rates in preterm infants (Gui et al. 2019), and have identified microstructurally altered brain white matter networks in the associative and limbic cortico‐basal ganglia‐thalamocortical circuits, involving the dorsolateral prefrontal cortex, the orbitofrontal cortex and the amygdala (Fischi‐Gomez et al. 2014; Cismaru et al. 2016). These brain structural changes were coupled with deficits in early emotion processing and emotion regulation, as well as attention, executive control and social reasoning difficulties. More recent data from brain lesion‐free preterm newborns provide evidence for further altered salience (anterior insula to anterior cingulate) network functionality already in the newborn period, a network that allows to adapt behavior according to the predictive value of stimuli, positive (reward) or negative (punishment). Predictive relations between stimuli and outcome are learned through experience, and preterm infants clearly have very different early life experiences with extreme situations of non‐predictability. Salience has been shown to be crucially involved in neuropsychiatric disorders seen in adults born preterm. These findings raise the question of how to induce resilience through more predictable stimuli in the newborn period. A recent research project has engaged in introducing interventions in the newborn period by maternal voice and music to introduce meaningful multisensory stimuli to the newborn environment (Fig. 8; Lordier et al. 2019a). The personalized music interventions to preterm infants have been shown to restore the functional connectivity between the salience network and auditory, sensorimotor, frontal, thalamus and precuneus networks, resulting in brain networks organization more similar to that of full‐term infants, suggesting that music prevents deficits in brain circuitry in preterm infants and may improve future socio‐cognitive function (Lordier et al. 2019b).

It is now well established that the environment and the neurosensory stimuli have an impact on sensorimotor, cognitive and psychological development, particularly during the neonatal period, and fMRI and EEG are valid methods to study early functional competence of the developing brain (Adam‐Darque et al. 2017; Lordier et al., 2019a, 2019b). Only just emerging though is the impact of sensory stimuli and general cellular activity on interneuron survival and distribution within the cortex (Denaxa et al. 2018; Wong et al. 2018). This topic is worth further exploration, as some interneuron populations are much more numerous in human prefrontal cortex compared with rodent model organisms (Lim et al. 2018).

## Conclusion: how can these new approaches contribute to our mechanistic understanding of disease etiology?

The elucidation of the mechanistic underpinnings of a given neuropsychiatric disorder requires detailed knowledge of the ultimate phenotypic outcome of that disorder, the normal developmental processes that are subverted as the disease progresses, and the environmental, genetic or epigenetic insults that contribute to establish the disorder. In the last decade, we have made substantial progress identifying the brain regions and cell types affected by many neuropsychiatric disorders; begun to uncover the cellular diversity and processes that underlie cell division, differentiation and maturation in different regions of the brain; and identified numerous common and rare genetic variants, including single nucleotide polymorphisms, copy number variants and de novo mutations associated with disorders including Alzheimer's disease, schizophrenia, bipolar disorder and ASD. These advances have been greatly facilitated by new technologies, including improved and more cost‐effective sequencing, and the development of new model systems, such as the development of 2D and 3D organoid cultures. In particular, although post mortem tissue will likely remain essential for many analyses, organoid cultures allow the observation and manipulation of many aspects of human brain development with nearly unlimited temporal resolution and fewer ethical and practical concerns. Similarly, the ability to conduct longitudinal neuroimaging studies of both the neurotypical and diseased human brain will offer increased resolution concerning the timing, phenotype and variability of disease onset and progression. As a consequence, our ability to understand neurological and psychiatric disorders and potentially generate therapeutic remedies is likely to continue to accelerate.
